# Investigating PTG in Cancer Patients: The Role of Time Dimension in the Experience of Personal Growth

**DOI:** 10.3390/ijerph19159619

**Published:** 2022-08-04

**Authors:** Chiara Fioretti, Viola Vinciarelli, David Faggi, Lucia Caligiani, Francesca Tessitore, Gianluca Castelnuovo, Mauro Cozzolino

**Affiliations:** 1 Department of Human, Philosophical and Educational Sciences (DISUFF), University of Salerno, Via Giovanni Paolo II 132, 84084 Fisciano, Italy; 2 Psycho-Oncology Unit, USL Toscana Centro, Via Antella, 58, 50012 Bagno a Ripoli, Italy; 3 Department of Psychology, Catholic University of Milan, 20123 Milano, Italy; 4 Psychology Research Laboratory, Istituto Auxologico Italiano IRCCS, Largo Agostino Gemelli, 1, 20123 Milano, Italy

**Keywords:** post-traumatic growth, cancer, time dimension, illness experience, narratives

## Abstract

This study explored the experience of growth related to being a cancer patient by implementing a thematic analysis. An online questionnaire was completed by 69 patients narrating their growth experience related to cancer. Collected narratives were analyzed by running a deductive thematic analysis, starting from the five domains of the Post-Traumatic Growth Inventory (PTGI) and searching for the presence or absence of topics. Descriptive statistics and correlational analysis were performed. The five factors of the PTGI were identified in the narratives. The thematic analysis we performed defined a further theme that we labeled the “time dimension”, which saturated 37.7% of the entire sample. The presences of four sub-themes related to the “time dimension” were also found: “tracing a new temporal rhythm”, “the value of deserved time”, “facing the caducity of life” and “a view on the future”. Each sub-theme significantly correlated with the theme of the “time dimension”. This emergent theme does not correlate in our results with other domains of personal growth in cancer previously described in the scientific literature, emerging as an independent variable not significantly associated with other domains of post-traumatic growth. Our results suggest further investigation in the role of the time dimension in the practical and emotional experience of growth with regard to cancer.

## 1. Introduction

Several authors have observed and defined a process of personal development after an extremely stressful event [1]. The dominant model of post-traumatic growth is that of Tedeschi and Calhoun [2], who defined it as a “positive psychological change experienced as a result of the struggle with highly challenging life circumstances or traumatic events” [3] (p. 1). The authors hypothesize that when a traumatic experience occurs, a series of schematic structures and basic beliefs that normally allow the subject to generate a sense of security and meaning in one’s existence and to make assumptions about the future and how to move toward that future are altered or destroyed [2,4]. The psychological disturbance given by trauma is necessary for the triggering of the growth process and Calhoun and Tedeschi [5] suggest that a good way to judge whether an event can be considered traumatic is to consider how it interrupts the life trajectory of an individual. The struggle with traumatic events can lead to a new life story with a before and an after, where the turning point is the traumatic event [6]. Thus, following a traumatic experience the individual must leave old beliefs and previous assumptions about the world, other people, and themselves in order to formulate new beliefs, goals, and identities that incorporate the trauma experienced [7]. In this conceptualization, post-traumatic growth (PTG) occurs if individuals, in the post-traumatic period, shed or give up certain basic goals or assumptions, while continuing in the attempt to construct new schemes, goals, and meanings. The construct of post-traumatic growth identifies a prolonged period of time, from days to years, in which people develop new ways of feeling, thinking and behaving, and experiencing personal and existential growth rather than a return to pre-traumatic levels of functioning [8]. Thus, growth is seen as an outcome in itself, but also as a process.

In the mid-1990s, based on available literature and interviews conducted with individuals who had experienced trauma, Tedeschi and Calhoun [9] identified three general areas in which a growth outcome occurred: changes in relationships with others, philosophy of life, or view of one’s self. After that, with the development of the Post-Traumatic Growth Inventory (PTGI), five major domains emerged through factor analysis, namely, “appreciation of life,” “personal strength,” “new opportunities,” “relationships with others,” and “spiritual change” [10]. The PTGI [10,11] is a 21-item questionnaire that measures individual perceptions of positive change following a traumatic experience. Subjects are asked to rate on a Likert scale the extent to which they have changed positively in each of the PTG domains identified by Tedeschi and Calhoun [10].

Regarding the five domains of the PTGI, greater appreciation for life and a change in personal priorities are common experiences in those coping with traumatic experiences. Typically, greater importance is placed on the simpler “little things” that were previously taken for granted, whereas what was considered important before the trauma is no longer as salient afterward [5]. It appears that people become more aware of what they can do and what they can experience, rather than what they lack or cannot do. Confronting the traumatic event thus prompts a greater appreciation for the countless gifts of existence [12]. The domain of “personal strength” is summarized by the authors as follows, “I am more vulnerable than I thought, but much stronger than I ever imagined” [5] (p. 5). The general sense of greater personal strength (or recognition that one possesses it) is experienced as an increase in self-esteem, self-confidence, and as a perception of one self as a victor or survivor (rather than a victim; ibid.). This can lead to behavioral changes, such as a commitment to learning something entirely new [13]. The domain of “new possibilities” can be experienced through the development of new interests, activities, or habits that would not have been undertaken had the triggering event not occurred [8]. At the relational level, not only the relationships themselves, but also the attitudes or behaviors within them, may change positively (ibid.) or reflect a conscious decision to communicate one’s affection for them and the value they possess more deeply [13]. In addition, subjects experience a greater sense of compassion, empathy, intimacy, closeness, and freedom to be themselves, exhibiting fewer interpersonal fears or concerns about being rejected [5]. Similarly, the decision to move away from relationships no longer seen as positive or beneficial may also take place and may also be related to changes in perceived personal strength [8]. Changes in the domain of spiritual and existential questions relate to a growth in philosophy of life (i.e., in a sense of greater purpose and meaning in existence) and perhaps also in greater clarity in the answers provided to fundamental existential questions [5].

A limitation of the PTGI is related to the fact that individuals report their growth experiences by having to respond within specific domains predetermined by the researchers, presumably not allowing for all areas of growth to be captured. In this sense, it is qualitative methodologies that can provide a detailed description and a deep and extensive understanding of individuals’ experiences of major life crises, allowing them to overcome that limitation of quantitative tools that does not allow them to capture all areas of growth or to capture the quality and centrality of the changes experienced by individuals [14]. In fact, when individuals report their growth experiences without having to respond within specific domains suggested by the researcher, we can be relatively certain that the responses provided are meaningful and relevant to the participants [15]. Thus, the need for a more detailed description and deeper understanding of individual growth experiences has emerged, which can be achieved through the use of qualitative research methodologies. The importance of qualitative research is also related to the fact that we can consider the narrative device as a space of meaning-making with respect to the traumatic experience, through which the narrator reconstructs an interrupted self-narrative [16,17,18,19].

Several studies have investigated PTG and its correlates in cancer patients [20,21,22,23,24,25,26,27,28,29]. The existential fracture following a cancer diagnosis [20,21] is linked, at least in part, to uncertainty and fear towards the future, which according to Cordova [30] can lead patients towards a different vision of life and a re-evaluation of daily priorities. Even the fact that the path of the cancer patient lacks an identifiable beginning and end that define the trauma temporally could be a determinant element of the specificity of the growth process of the cancer patient.

As reported by Sumalla, Ochoa, and Blanco [31], several authors [32,33,34] have identified substantial differences between stress characterizing the cancer patient experience and that associated with other acute adverse events. First, acute traumatic events usually affect the subject from the outside, whereas a characterizing factor of oncologic disease is the internal nature of the cancer, which proliferates in the patient’s body. Moreover, while for many traumatic events it is possible to identify a single traumatic stressor, in the case of cancer disease we are in the presence of a chronic stress linked to different sources (e.g., diagnosis, severity of the disease, prognosis, side effects of treatments, etc.) and of which it is not easy to clearly establish a beginning and an end. Therefore, unlike in acute trauma where the repetition of symptoms is associated with a past traumatic event, in the course of cancer disease most of the intrusive cognitions presented by the patient consist of fears for the future.

There has been a great deal of research on PTG in the field of oncology over the past twenty years, but the vast majority of studies have used quantitative methodologies, particularly the PTGI [27]. In Hefferon, Grealye, and Mutrie’s (2009) [35] systematic review of qualitative studies conducted in the context of physical pathology, we note that only four of the research studies designed to specifically investigate PTG explored the construct with purely qualitative research designs, and of these four only one was aimed at cancer patients. The study by Hefferon et al. (2009) [35] found that recovery from cancer illness could lead to a new awareness and importance of the body, revealing a potentially new element in the PTG process for trauma resulting from cancer illness. Furthermore, a recent qualitative study on PTG in cancer proposed the presence of peri-traumatic growth in patients experiencing the active phase of disease [36]. As we see here, qualitative tools allow us to analyze underdeveloped areas of research and also to understand the differences that may exist between growth experiences triggered by different traumas.

Starting from the scientific literature and gaps in knowledge about specific aspects of PTG related to a serious and chronic disease, the present study aims to explore narratives in the PTG experience of people suffering from oncological disease by implementing a thematic analysis. Starting from the five domains of the Post-Traumatic Growth Inventory, we analyzed narratives in order to investigate:

(1) If the defined domains saturated participants’ experiences as collected by narratives and how participants’ experiences related to each of them;
(2) If other domains of growth emerged with consideration to the specific context of oncological disease.

Thus, the general aim of the study was to explore the qualitative experience of growth related to being a cancer patient in depth, defining which domains are reported in narratives.

## 2. Materials and Methods

### 2.1. Participants

The participants consisted of 69 people suffering from cancer (67 females and 2 males) who voluntarily participated in the study. The inclusion criteria for the present study were receipt of a cancer diagnosis, being able to write in Italian, and being more than 18 years old. The mean age of participants was 48.42 years (SD = 9.45). Of the participant population, 52% (*n* = 36) were undergoing curative therapies (as for instance chemotherapy, radiotherapy or immunotherapy), while 48% (*n* = 33) were undergoing follow-up checks and other surveillance and prevention therapies (such as hormonal therapy). The mean time spent from diagnosis in the sample was 43.5 months (SD = 55.3).

### 2.2. Procedures and Measures

Narratives were collected by means of an online survey between October and December 2019 distributed by the website of Tages Charity, which collaborates with the Psycho-Oncology Unit of the *** Health Service in a city in central Italy and provides support and services to research and intervention projects in the fields of psychology and psychotherapy. Ethical approval was given by the ethical committee of the same charity (protocol reference number: 03-2019/260819). The data were collected in a manner that preserved anonymity and in line with the ethical principles for research involving human subjects promoted by the Helsinki Declaration.

After a brief questionnaire concerning demographic and medical information in order to define the presence of inclusion criteria, an online informed consent form was signed by each participant.

Afterward, a narrative task was proposed to participants to investigate narratives of post-traumatic growth in oncology: “In order to deepen the topic of our research we kindly ask you to think about your experience of illness. If you believe that your experience can be associated—among many other issues—with a growth you have perceived in your life, we ask you to tell us about your experience. You can tell us your thoughts by letting them flow, also by having recourse to specific memories or events that have transmitted to you the feeling of personal growth linked to the experience of oncological disease. There are not time or space limits for your narration. Once again we thank you for your precious testimony”. No limitations of time and space were given to participants to complete their narrative; they could leave the task if and when they wanted. None of them refused to participate or interrupted the task while narrating. The e-mail address and institutional phone number of the main researcher were communicated to all participants to establish a line of contact for further information on the study and its results if needed.

### 2.3. Data Analysis

In accordance with the goals of the study, the collected narratives were analyzed by running a deductive thematic analysis as described by Braun and Clarke (2006) [37]. This type of analysis starts with a specific theoretical framework that constitutes the scientific basis for approaching the data and guides the search for the presence or absence of topics [38]. It is, in short, a “data-driven” analysis (Braun and Clarke, 2006). After each researcher’s individual familiarization with the data, the analysis required a lengthy process of group thought and active dialogue among the researchers regarding the themes that emerged throughout the research [37]. In our study, the theoretical approach at the base of the data analysis was the construct of post-traumatic growth as defined by Tedeschi and Calhoun (1996) [10] in their construction and description of PTGI.

For the present study, a junior researcher-in-training and an older researcher with experience in narratives of illness performed data analysis. A pilot sample of 20 narratives was carefully and individually read by the two researchers, who identified the presence or absence of PTG factors as defined by Tedeschi and Calhoun (1996) [10]. Researchers then calculated the K of the Cohen index of agreement of the coded themes. The index was fully acceptable (K = 0.87). The main sample was therefore analyzed by a single researcher, who identified the presence of PTG factors and/or coded new themes when and if present in each narrative.

At the end of the data coding procedure, a final discussion of data was performed in a group setting involving all research group members (author 3 and author 4).

After defining the emergent themes in the narratives, data were coded as 0 when a theme was absent and 1 when it was present in a narrative. The non-parametric Spearman’s rho test was run in order to find potential associations among emergent themes in the narratives. Descriptive statistics and correlational analysis were performed by means of IBM SPSS statistics software.

## 3. Results

As described in Table 1 (Table 1), the five factors of post-traumatic growth as highlighted by Tedeschi and Calhoun (1996) [10] were identified in the narratives.

“Appreciation of life” and “personal strength” were the most-reported factors, and represent 53.6% and 52.1% of the collected narratives on post-traumatic growth, respectively. Furthermore, 39.1% of narratives reported experiences of growth in interpersonal relationships, as well as the discovery of new opportunities in life in 21.7% of cases. Lastly, 0.05% of narratives were related to the experience of spiritual changes due to suffering from oncological disease.

### 3.1. Time Dimension

The thematic analysis performed defined a further theme that we labeled as the “time dimension” that saturated 26 narratives out of 69 (37.7% of the whole sample). A change in the experience of the flow of time was reported by 26 participants, in addition to being aware of the value of time in their lives.

Participant #35, for instance, narrated:

*“[…] When you live the disease on your skin you realize that everything else doesn’t matter. I mean, I live day by day […]. Yes, I do. Because when you have dealt with the beast every day of your life for a while, then every month, every year, for you is like newfound time […]”.*

This man, experiencing an oncological disease that started 12 months prior, described the feeling of perceiving a new dimension of time in which living day by day had the meaning of tracing a new temporal rhythm of life.

Similarly, participant #38 reported:

*“We [she and her family] have learned to live intensely every day, because life is so fast! Every moment of life is fragile and wonderful […]”.*

In this narrative, time spent, both in days and single moments, acquired the quality of being wonderful but fragile. Thus, time in people living with cancer can be a dimension characterized by caducity, forcing patients to fully experience every moment of their lives.

While performing the thematic analysis, researchers conducted a group discussion of the presence of four sub-themes related to the time dimension that described all the coded narratives.

Table 2 shows the four sub-themes and the occurrence of each in the narratives (Table 2).

#### 3.1.1. Tracing a New Temporal Rhythm

The first emerged sub-theme was labeled “tracing a new temporal rhythm” and concerns the experience 11 participants reported of finding a new rhythm of life characterized by greater awareness about the importance of every single moment and day in life after being diagnosed with cancer. In particular, some participants shared their feelings of having to run and run without stopping before diagnosis and giving importance to many instants of their life. Thus, time acquired a new dimension in their experience, demonstrating a growth in their way of enjoying life and its happenings.

Some participants reported the relevance of each moment and their attempts to slow down in order to live each day granted to them more fully. Indeed, we received reports that after the trauma, the passage of time in life was savored without underestimating days, months, or years. In this sense, time expanded and became denser, with participants experiencing the present day by day, such that every moment of the “time of life” was worthy of being lived and appreciated.

The following narrative provides an example of the sub-theme:

*“I learned to capture moments and seconds and taking pictures in my mind to remember my family united and happy. I’m always so scared about cancer, but I live day by day and I enjoy every smile life gives me […]”.*

(Participant #8)

The reference to moments, seconds, days, and years was constant in the narratives. In this specific sub-theme, it is related to the need to enjoy as much time as possible with a new rhythm related to the experience of living slowly.

Participants #18 summarized this experience, narrating:

*“What has really changed in me is my perception of time. I mean, many things are changed and I really feel a different person. But cancer has certainly changed my priorities and firstly my perception of time passing”.*

#### 3.1.2. The Value of Deserved Time

Moreover, 8 participants reported a feeling of having discovered the value of time deserved for oneself. The second sub-theme, named “the value of deserved time”, refers to the fact that personal growth implied the re-evaluation of the activities that deserved to have time spent on them.

Patients reported re-evaluating the time they devoted to themselves and their attempts to find time that was previously “missing”. In their narratives of growth, participants shared reflections on a lack of time before the disease and described how they re-defined and re-organized life activities according to new perceived needs.

The following narrative provides an example:

*“Through illness, I have learned to give me time too; time that before I didn’t have, that most of times I should have “steal” from other more important things. Now I have no alternative: what has to be done for me, I must find the time for it”*.

(Participant #6)

Similarly, participant #25 stated:

*“At the beginning I felt the strong impulse to always have to do something, almost a sense of suffocation if I didn’t do something. I never had time, because I had to do, do and do, thinking that life is short. Then I started to spend my energy by trying to use it in a constructive way and devoting more time to myself. I asked for part-time at work, I devoted more time to children, myself and my partner. I feel that time belongs to me now”.*

Both narratives refer to growth as a process of awareness of deserving more time for oneself. Growing with and after cancer means learning to give oneself more time for important matters and less for other, less consistent activities.

#### 3.1.3. Facing the Caducity of Life

The third sub-theme that emerged, reported by 5 participants, was labeled “facing the caducity of life” and refers to patients’ feeling that cancer disease gives themselves a deadline to their time in life. In participant narratives, oncological disease pushed participants to face the caducity of life and to re-think and perceive time in a different way with awareness of the fact that “there will necessary be an end” (Participant #57). Participants perceived a change in the time dimension due to the possibility of dying.

In particular, a female participant (#18) narrated:

*“What has really changed in me is the perception of time. Facing with the possibility of dying, I suddenly realized how many things I would wanted to do and say and it helped me to grow”.*

Another participant (#49) argued:

*“It is true that the second life begins when you realize you only have one. Time is now!”*

Again, the feeling of time flowing and the finitude of life helped some patients to experience a personal growth related to a new perception of the importance of time enjoyed and fully managed.

#### 3.1.4. A View on the Future

The fourth and last sub-theme of the time dimension deals with “a view on the future” and was reported by 3 participants in their narratives. Personal growth after cancer-related trauma led these participants to consider time not just as the present but also as new opportunities for the future. Biographical disruption due to cancer is not only related to uncertainty and fear towards the future, but also the need to perceive the future as a time for oneself and to re-evaluate new life projects.

Participant #50 narrated:

*“The disease led me to think some really important things I simply was used to connect to a time far from me. Will I see my daughter in her white dress? It’s all uncertain now, but I found the strength to imagine future time”.*

Again, the time dimension was connected with post-traumatic growth in these narratives, but differently from other sub-themes, it was related with the opportunity to think about the future and not re-schedule and re-organize the past and the present. In this sense, in our results, the future seems to be a temporal dimension correlated with the experience of growth in cancer.

To investigate the potential association between the five factors of PTG as defined by Tedeschi and Calhoun (1996) [10] and the theme of “time dimension” and its four emerging sub-themes, non-parametric correlations were implemented. The results of Spearman’s rho test show no correlations between the theme of the “time dimension” and the five factors of the construct of PTG. (Table 3).

Considering the four sub-themes, Spearman’s rho test shows a significant non-parametric correlation between “tracing a new temporal rhythm” and the factor “appreciation of life” (rho = 0.405; *p* < 0.01). Other sub-themes did not significantly correlate with the five factors of PTG.

Each of the four sub-themes significantly correlated with the emerging theme of the “time dimension”.

## 4. Discussion

The present study aimed to investigate post-traumatic growth in a sample of 69 oncological patients by means of a deductive thematic analysis [37,38]. This qualitative analysis method starts from a specific theoretical framework and approaches data with the aim of investigating the presence or absence of topics reported in the scientific literature [38]. In our study, this “data-driven” analysis approach was performed based on the construct of post-traumatic growth as defined by Tedeschi and Calhoun (1996) [10] in their construction and description of the Post-Traumatic Growth Inventory.

Starting from the five factors of the inventory, our research group analyzed the collected narratives to find if the defined domains saturated participant experiences and if, considering the specific context of oncological disease and its peculiar experience of suffering, other themes emerged.

Overall, all five factors defined by Tedeschi and Calhoun (1996) [10] were reported by the oncological patients of our study, with a prevalence of the “appreciation of life” and “personal strength” domains. The recent systematic review on PTG in cancer patients by Casellas-Grau, Ochoa, and Ruini (2017) [27], despite assessing 76% of participants using the PTGI, did not report the prevalence of the five factors and only considered the total score of the inventory.

Nevertheless, considering the study by Brunet and colleagues (2010) [39] on an examination of the factor structure of the PTGI among breast cancer survivors, our results are in line with the factor saturation found by the authors, with a higher presence of “appreciation of life” and “personal strength” and a lower presence of “spiritual change”.

Our results point out a further emerging theme that has not yet been considered by the scientific literature regarding PTG in cancer: the time dimension. In participants’ narratives, time and growth were related in 37.7% of the sample. Particularly, patients narrated different ways of rethinking, reorganizing, and resetting their values as well as the dimension of time in their lives due to the experience of growth related to being a person suffering from cancer. Nevertheless, although 26 patients reported the time dimension in their narratives, we found that this emergent theme did not correlate with the five factors of PTG described by Tedeschi and Calhoun (1996) [10] or with other domains of personal growth in cancer found in the scientific literature so far [27].

Looking at the narratives and at the results of non-partial correlations, the time dimension seems to emerge as an independent variable not significantly associated with other domains of PTG.

This datum suggests the need to further investigate the role of the time dimension in the practical and emotional experience of growth in cancer.

In effect, there is a lack of evidence on the meaning of time and its elaboration across the oncological experience, and the concept of time has not yet been related to that of personal growth. Our results show that more than one out of three patients evidenced and described the time dimension as an important part of their post-traumatic growth.

In particular, four sub-themes emerged in and saturated our study regarding the time dimension of PTG: “tracing a new temporal rhythm”, “the value of deserved time”, “facing the caducity of life” and “a view on the future”. Each of them brings a specific issue connecting time and PTG to the discussion: from the awareness of needing a new rhythm of life to that of deserving more time for oneself, and from the rise of new opportunities due to having experienced the finiteness of life so strongly to the birth of a view of the future. Again, the majority of these issues were not correlated with PTG as defined by Tedeschi and Calhoun (1996) [10] in their PTGI. A significant non-partial correlation was found between the sub-theme “tracing a new temporal rhythm” and the factor “appreciation of life”. Looking at the items of the PTGI, among the three of them referring to the “appreciation of life” domain we find the item “appreciating each day” [10] (p. 460). This statement can be partially related to the attempt of cancer patients to trace a new rhythm of life by discovering the meaning and the value of each day of their lives. Nevertheless, the found sub-theme embraces other nuances of growth related to the time dimension that within our opinion are not well represented by a single item. For instance, participants reported learning to enjoy life’s happenings and discovering a new way of living instead of the “run run” rhythm they experienced before the disease. In this sense, growth in terms of the time dimension does not just involve a better appreciation of life; rather, it is linked to a behavioral change in terms of starting to live “slowly” and enjoying the little moments life offers.

Considering the other three sub-themes, no significant correlations were found with the five factors of PTGI.

Again, our results suggest the need for further investigation of the meaning of time dimension and potential changes in the way oncological patients experience it.

## 5. Conclusions

To conclude, our results bring one possible issue that is as of yet uninvestigated to the discussion regarding insights on PTG and cancer. Further studies could deepen the meaning of time in oncological disease and how it relates with both distress and growth/resilience in patients. Our results introduce interesting issues to be considered in clinical intervention with cancer patients in every stage of the care path. Psychological intervention should investigate the meaning of time and its change across the illness experience as a variable related to potential personal growth and to the definition of disease as a stressful but also growing part of one’s life path. Currently, most clinical interventions are in fact focused on stress reduction and trauma investigation, omitting the potential growth related to suffering from cancer.

A limitation of the present study is that we did not consider the potential role of different kinds of cancer diagnoses on PTG. The dimension of time could be linked more with a critical oncological diagnosis and could acquire a deeper meaning in patients facing advanced stages of disease. Similarly, another limitation was that we did not consider the time from diagnosis as a potential predictive variable for the occurrence of the time dimension in narratives regarding PTG. The sample size could be expanded in the future to better control this variable. Furthermore, our sample was composed of 67 female patients out of 69. Further studies will balance the gender of the participants in order to consider potential differences in cancer patients’ PTG due to this variable. Similarly, the potential role of patient age in the presence of the time dimension in the experience of PTG related to cancer could be also investigated.

## Figures and Tables

**Table 1:** Presence and absence in collected narratives of each factor of PTG as described by Tedeschi and Calhoun (1996).

	Presence	Absence	N
Personal strengths	36	33	69
Appreciation of life	37	32	69
New opportunities	15	54	69
Relationships with others	27	42	69
Spiritual change	4	65	69

**Table 2:** Presence and absence in collected narratives of each sub-theme of the time dimension theme as they emerged from thematic analysis performed by researchers.

	Presence	Absence	N
Tracing a new temporal rhythm	11	58	69
The value of deserved time	8	61	69
Facing the caducity of life	5	64	69
A view on the future	3	65	69
Total occurrence in narratives	-	-	26
Co-occurrence of sub-themes in narratives	-	-	1

**Table 3:** Non-parametric correlations with Spearman’s rho test among the five factors of PTG (Tedeschi and Calhoun, 1006) and the time dimension and its four emerging sub-themes.

	Personal Strengths	Appreciation of Life	New Opportunities	Relationships with Others	Spiritual Changes	Time Dimension	Tracing a New Temporal Rhythm	The Value of Deserved Time	Facing the CaduCity of Life	A View on the Future
Personal strengths	______	−0.180	0.223	0.0540	0.113	−0.094	−0.138	−0.070	0.044	−0.800
Appreciation of life		________	0.067	0.091	0.106	0.183	0.405 **	0.059	0.148	−0.087
New opportunities	0.223	−0.180	_________	0.225	−0.131	0.047	0.058	0.136	0.124	−0.0112
Relationships with others	0.0540	0.091	0.225	_________	-0.072	−0.011	−0.025	0.182	0.005	−0.120
Spiritual changes	0.113	0.106	−0.131	−0.072	_____	0.063	0.061	0.103	−0.069	−0.053
Time dimension	−0.094	0.183	−047	−0.011	0.063	______	0.560 ***	0.479 **	0.244 *	−0.274 *
Tracing a new temporal rhythm	−0.138	0.405 **	0.058	−0.025	0.061	0.560 ***	______	0.087	0.031	0.101
The value of deserved time	−0.070	0.059	0.136	0.182	0.103	0.479 **	0.087	______	0.072	0.144
Facing the caducity of life	0.044	0.148	0.124	0.005	−0.069	0.244 *	0.031	0.072	______	0.077
A view on the future	−0.800	−0.087	−0.112	−0.120	−0.053	−0.274 *	0.101	0.144	0.077	____

*: *p* < 0.05; **: *p* < 0.01; ***: *p* < 0.001.

## Data Availability

Regarding data that contain potentially identifying or sensitive patient information, access to such data would be allowed in forms judged by the Ethical Committee to not cause any breach of anonymity or privacy of patient data. Data are available from the Tages Charity and the Psycho-Oncology Unit of USL Toscana Centro for researchers who meet the criteria for access to confidential data. Please contact the Office of the Psycho-Oncology Unit or its director Dott. Lucia Caligiani to access data.
